# Evaluation of Three Human-Use Glucometers for Blood Glucose Measurement in Dogs

**DOI:** 10.1155/2022/9112961

**Published:** 2022-11-24

**Authors:** Matheus Albuquerque Basilio dos Santos, Alesssandra Martins Vargas, Paula Nunes Rosato, Carolina Gomes Andrade, Camila Marinelli Martins, Giuliana Petri

**Affiliations:** ^1^Metropolitan University of Santos (UNIMES)-Prefeito Antônio Manoel de Carvalho Avenue, 3935 Morro Nova Cintra 11080-100, Santos, Sao Paulo, Brazil; ^2^College Anclivepa Ulisses Cruz Avenue, 285 Tatuapé 03077-000, Santos, Sao Paulo, Brazil; ^3^Anclivet Veterinary Laboratory Goias Street, 118, Gonzaga 11050-100, Santos, Sao Paulo, Brazil; ^4^São Judas Tadeu University Comendador Martins Street, 52, Vila Matias, 11015-530, Santos, Sao Paulo, Brazil; ^5^AAC&T Research Consulting LTDA Domingas Vigo Zaninelli Street, Boa Vista 82540-096, Curitiba, Parana, Brazil

## Abstract

**Background:**

Glucometers or portable sensors are used to quickly measure blood glucose at low cost. They are used in veterinary practice and by guardians to monitor diseases that require, as in diabetes mellitus. However, not all commercially available glucometers (human and veterinary) are suitable for this purpose. *Hypotheses/Objectives*. The objective was to evaluate the analytical and clinical precision of three human-use portable glucometers. *Animals*. This study evaluated 115 samples in three glycemic ranges (hypoglycemia, normoglycemia, and hyperglycemia) from 82 dogs recruited from veterinary services.

**Methods:**

The portable glucometers are the FreeStyle Freedom Lite®, FreeStyle Optium Neo®, and On Call Plus® models. Glucometer results were compared with the enzymatic colorimetric glucose oxidase laboratory reference method. Using descriptive and comparative statistical analysis, there were correlations between these devices and the standard method, ISO 15197 : 2003 and ISO 15197 : 2013 standards, and error grid analysis.

**Results:**

Only the Freedom Lite® device observed a statistical difference when compared with the reference method. Despite the underestimated glucose concentrations assessed with humane devices, all three tested herein showed a positive coefficient. However, none of these achieved all ISO guidelines. *Conclusion and Clinical Importance*. Although there was wide use of portable humane devices for dog glucose measurements on routine, the results are generally inferior when compared to the reference method. The FreeStyle Optium Neo® glucometer obtained the best result and is therefore the best option among the glucometers evaluated; however, for the first attendance on veterinary routine, all three glucometers had a satisfactory glucose measurement until the reference method availability.

## 1. Introduction

Glucometers or portable sensors are devices used to measure blood glucose quickly, easily, and at low cost in both humans and animals [1]. The small sample volume required and the generation of immediate results are important advantages over automated analyzers [2]. Portable glucometers developed for human use are widely adopted in veterinary practice; however, they should be validated for adequate use in animals, whereas such devices are developed for humans [3]. Likewise, glucometers developed for veterinary use may not provide reliable results [3–5]. An inadequate device can result in an incorrect hypoglycemia diagnosis and lead to inaccurate glycemic control [6] and may be used for decision-making for adjustments in insulin therapy for diabetic patients [2].

In general, portable glucometers underestimate blood glucose concentrations compared to the standard method [3, 7]. The higher the hyperglycemia, the greater this difference [2]. Furthermore, samples may suffer interference from conditions such as hematocrit concentration (the lower the hematocrit level, the greater the blood glucose reading on the glucometer, and vice versa) [5]. Other interferences are sample volume, time, test strip stability, altitude, hemolysis, blood temperature, humidity, partial oxygen pressure in blood samples, prandial state, and the type of sample used (venous, arterial, or capillary blood) [1, 2, 8].

Due to the large number of portable glucometers available commercially, studies evaluating such products are essential. This supports the use of reliable glucometers to measure blood glucose concentration in dogs [5]. The efficiency evaluation of portable glucometers used to be performed by comparing the glycemic results obtained using glucometers in tests with the values generated by the glucose oxidase or hexokinase reference methods [1, 3, 9, 10].

Thus, the aim of this study was to evaluate the performance of three portable glucometers for human use, in different dog's blood glucose ranges (hypoglycemia, normoglycemia, and hyperglycemia) and to compare with the enzymatic colorimetric glucose oxidase method (reference test) in animals with normal hematocrit concentration.

## 2. Materials and Methods

### 2.1. Animal Inclusion Criteria

The experiment was performed between November 2018 and April 2019, and the animals were recruited from the “*Universidade Metropolitana de Santos*”-Veterinary Service and other regional veterinary clinics located in Santos City, Sao Paulo, Brazil. All owners agreed and signed the authorization for sample use. Were included dogs over six months of age, both sexes, diagnosed with diabetes or another endocrine pathology or yet healthy and who were not hospitalized.

### 2.2. Glucometers and Reference Method

Whole blood was used to measure glucose concentrations with glucometers and plasma to glucose oxidase enzymatic colorimetric method analyses. A total of 180 blood samples were collected from 138 dogs, and after inclusion criteria use, 115 samples from 84 dogs were maintained.

The glucometers used herein were developed for human practice and were calibrated following the manufacturer's instructions. Weekly calibrations were performed when started a new test strip bottle. Three units of each glucometer (triplicate) were assessed, in which a total of nine sensors were used. The evaluated models were FreeStyle Freedom Lite® (Abbott Diabetes Care Inc.) (coulometric electrochemical sensor; glucose dehydrogenase measurement method); FreeStyle Optium Neo® (Abbott Diabetes Care Inc.) (amperometry assay; glucose dehydrogenase measurement method); On Call Plus® (TaiDoc Technology Corporation) (coulometry assay; glucose oxidase measurement method). In other words, each sample was measured with the same glucometer three different times after the sample collection due to the literature hypothesis that time after blood sampling could interfere with results. All measurements were performed up to four hours after the blood sample.

Blood samples were collected by venipuncture and were distributed as follows: tubes containing disodium ethylenediaminetetraacetic acid (EDTA) with added fluoride; tubes with EDTA; gel bottles with clot retractors and approximately 0.5 mL of residual whole blood. The glucometers were divided into three groups (three units of each model): group one (FreeStyle Freedom Lite®), group two (FreeStyle Optium Neo®), and group three (On Call Plus®).

All laboratory analyses were performed on the same day. Blood samples from the tube containing EDTA were used to obtain the hematocrit level by centrifuging whole blood in a microhematocrit centrifuge (CELM® model LS 3 PLUS), and reading was performed using a hematocrit card. The tubes containing fluoride EDTA and clot retracting gel were centrifuged to obtain plasma (up to four hours after the blood sample) and serum, respectively. The fluoridated plasma aliquot was used to measure plasma glucose concentration, and the serum sample was used to analyze the serum concentration of triglycerides and cholesterol. Plasma glucose concentrations and triglycerides and cholesterol measurements were performed using the GOD-trinder (glucose oxidase) and colorimetric (Enzymatic Trinder) methods, respectively. The readings were performed using commercial kits (Labtest®) and an automated biochemical analyzer (Labmax Plenno, Labtest®). Measurement of blood glucose was performed using the enzymatic colorimetric glucose oxidase reference method.

### 2.3. Blood Sample Exclusion Criteria

As recommended by the manufacturer of the automated biochemical analyzer, triglyceride concentrations ≥ 1,000 mg/dL may suffer significant interferences in laboratory blood glucose testing. Still, according to the information provided by the glucometer manufacturers, samples with cholesterol concentrations ≥ 500 mg/dL and triglycerides ≥ 3,000 mg/dL may be affected by the results. Therefore, samples that presented triglycerides ≥ 1,000 mg/dL, cholesterol ≥ 500 mg/dL, and hematocrit outside the 37 to 55% range were excluded. Glucometer results of blood glucose concentration that obtained on-quantitative readings (LOW or HI) were also excluded from this work, in addition to laboratory samples containing hemolysis, jaundice, or insufficient blood.

### 2.4. Statistical Analysis and Sampling

The sample size was determined using the ISO 15197 : 2013 guidelines, and this document determines that 100 samples should be collected and divided into different concentrations of glucose.

Descriptive analysis was performed with an estimate of the mean, median, minimum, maximum, interquartile range, and standard deviation of the quantitative variables. The final data were tested for normal distribution using the Shapiro–Wilk test, and the nonparametric approach was used (Shapiro–Wilk *p* value <0.001 for the three models). The correlation between glucometers and laboratory exams was evaluated using Spearman's correlation coefficient and the statistical difference between these using the Mann–Whitney *U* test. For better visualization of the data, scatterplots and boxplots were produced, comparing the glucometers and the laboratory exam. All tests were considered significant when *p* < 0.05, and these analyses were performed using SPSS 21.0 [11].

Accuracy between the glucometers against the reference method was assessed based on ISO 15197 : 2003 [12] and ISO 15197 : 2013 [13] standards. According to the ISO 15197 : 2003 [12] guidelines, 95% of results may vary up to 15 mg/dL in blood glucose concentration up to 74 mg/dL and have a maximum variation range of 20% for blood glucose ≥75 mg/dL. However, (stricter) ISO 15197 : 2013 [13] guidelines establish that 95% of the results vary up to 15 mg/dL in blood glucose concentration up to 99 mg/dL and have a maximum variation range of 15% for blood glucose concentration ≥100 mg/dL.

In addition, error grids with the glucometers and the glucose oxidase laboratory reference method were produced [14]. The error grid is divided into zones that represent the risk due to an incorrect measurement: zone A represents errors without clinical effects (analytical precision); zone *B* represents values that deviate more than 20% from the reference values, justifying changes in treatment, but without significant repercussions for the patient; zone *C*, values that can induce unnecessary treatment; zone *D*, the danger of serious errors in treatment; zone *E*, errors that can induce clinical conduct with dangerous consequences [10]. Error grid analysis is widely used in human medicine and categorizes measurements based on therapeutic consequences [1].

It was performed a Bland—Altman analysis to determine whether there was an agreement between tested glucometers and the reference method. Such an analysis estimates the differences between measurements and the mean difference, standard deviation, and concordance mean (CI 95%). Also, was used a Passing Bablok regression to evaluate the systematic difference between the standard method and the glucometers. Statistical significance was determined as 5%, and analysis was performed in *R* environment 4.0.4 (*R* Core Team, 2021).

To define a glucometer as precise, the ISO 15197 : 2013 [13] standard requires that 99% of the results be in zones *A* and *B* [7]. The error grid method was created in 1987 [15] and modified in 2000 [10, 14]. This analysis was performed in the *R* environment [16] using the “ega” package [17].

## 3. Results

Repeated blood glucose concentrations measured between glucometer models using the same blood sample compared with the reference method showed no statistically significant difference (Freedom Lite® *p* = 0.443, Optium Neo® *p* = 0.378, and On-Call Plus® *p* = 0.765). Laboratory plasma blood glucose ranged from 37 to 425 mg/dL.  Table 1 shows the blood glucose ranges assessed in this study.

**Figure 1:**
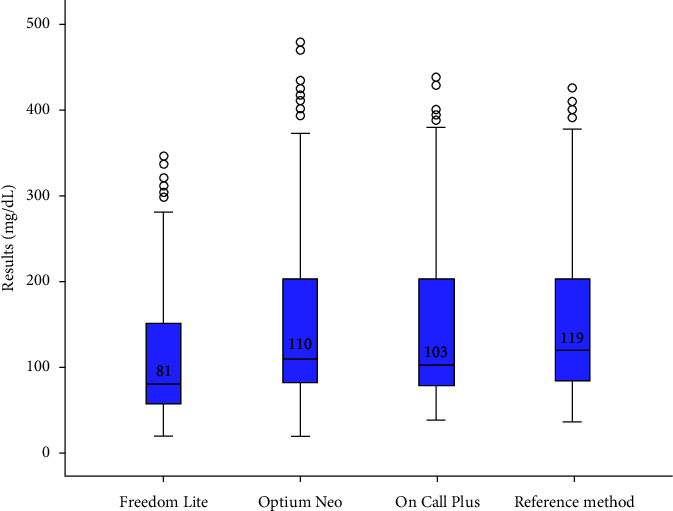
Boxplot of blood glucose values obtained using glucometers and the reference method in dogs.

There was no statistically significant difference between the results obtained with the Optium Neo® and On Call Plus® glucometers (*p* > 0.05) compared to the glucose oxidase reference method; however, there was a statistically significant difference between the values obtained with the Freedom Lite® model (*p* < 0.05) compared with the reference method (Table 2, Figure 1).

The three assessed glucometers underestimated the blood glucose concentration compared with the reference method. There was a smaller difference in the results obtained using the Optium Neo® model. A glycemic average of 158 mg/dL was observed using the reference method, and the averages with glucometers were Optium Neo® (151 mg/dL); On Call Plus® (145 mg/dL), and Freedom Lite® (111 mg/dL) (Table 2).

**Figure 2:**
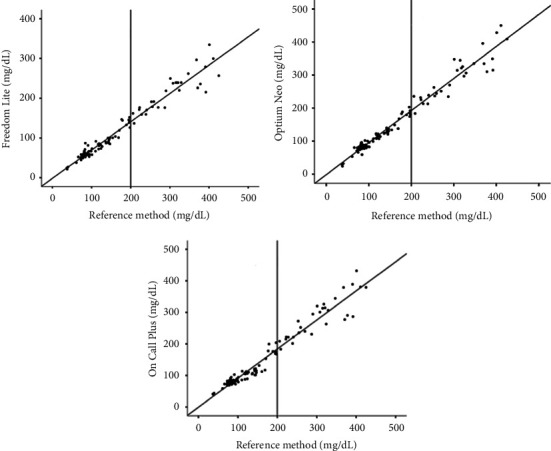
Graphics of glycemia dispersion obtained by the reference method (*x*) and the Freedom Lite, FreeStyle Optium Neo®, and On Call Plus glucometers (*y*) in dogs.

The three assessed glucometers obtained a good correlation when compared with the reference method. Portable device results had a positive coefficient, showing that the values moved in the same direction in relation to the reference method. However, graph analysis indicated that samples higher than 200 mg/dL had more significant dispersion between the glucometer results in relation to the reference method (Figure 2). Also, the results obtained with the Freedom Lite® model had more significant dispersion, especially in the higher blood glucose range.

**Figure 3:**
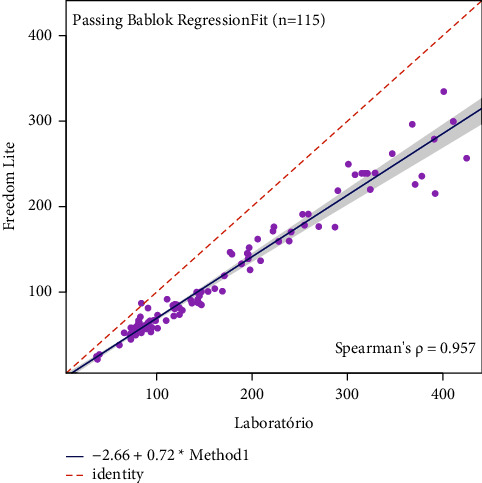
Passing-Bablok regression for the FreeStyle Freedom Lite® glucometer.

**Figure 4:**
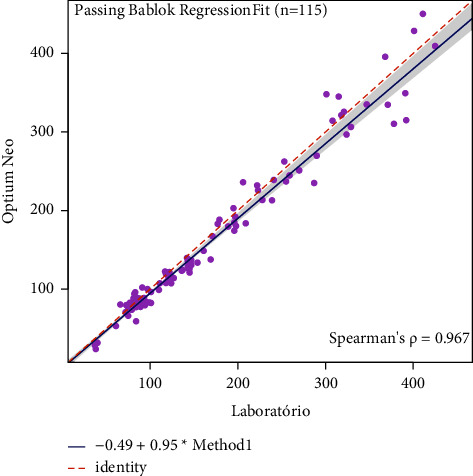
Passing-Bablok regression for the FreeStyle Optium Neo® glucometer.

**Figure 5:**
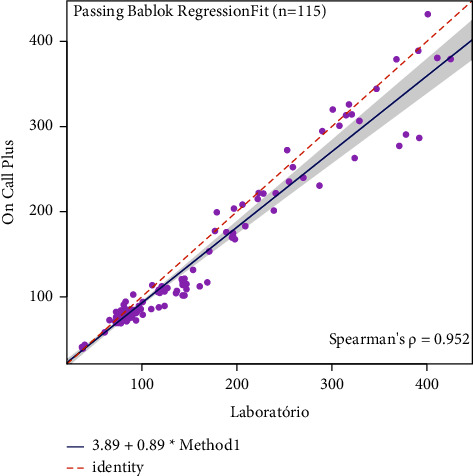
Passing-Bablok regression for the On Call plus® glucometer.

The concordance results were presented in Table 3. The Freedom Lite® glucometer measures 47,05 (estimated bias) less units than the reference method, with a significant statistical analysis observed in concordance limits (Table 3). The same results were obtained in the other two glucometers: the Optium Neo® measured a bias of 6, 23 and the On Call Plus® a bias of 13,08; both were of statistical significance. The passing-block regression was performed to visualize the proportional differences between the standard and glucometer results (Figures 3–5). All three systematic bias from glucometers did not differ significantly from the reference method.

Regarding ISO 15197 : 2003 [12], guidelines establish that 95% of glycemic results vary from 15 mg/dL to 74 mg/dL and have a maximum variation of 20% for blood glucose concentration ≥75 mg/dL. In this analysis, only the Optium Neo® glucometer reached the goal of 99.1% of the results in the established ranges. Regardless, the On Call Plus® model reached 85.2% of the results within acceptable limits and did not reach the minimum required. In turn, the Freedom Lite® glucometer reached only 12.2% of the results in the desired ranges (Table 4).

None of the evaluated glucometers achieved all of the ISO 15197 : 2013 [13] guidelines. These regulations establish that 95% of the results vary up to 15 mg/dL in blood glucose concentration up to 99 mg/dL and have a maximum variation of 15% in blood glucose concentration ≥100 mg/dL. The Optium Neo® model had the best performance, reaching 92.2% of the glycemic results in the established ranges. The On Call Plus® glucometer reached 79.1% of the values in the intended glucose ranges. The Freedom Lite® sensor achieved only 7% of the results in the ranges required by the ISO 15197 : 2013 [13] standard (Table 5).

**Figure 6:**
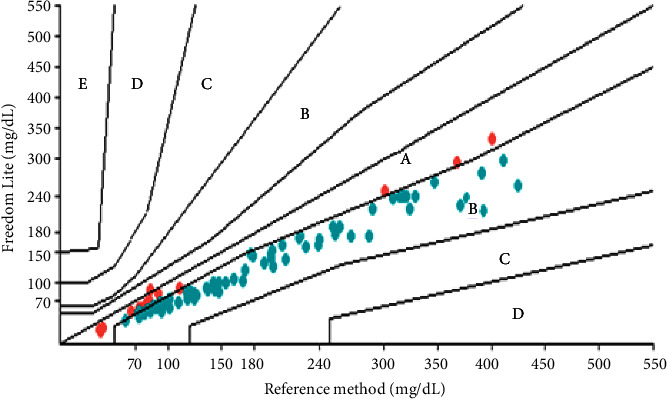
Error grid for the FreeStyle Freedom Lite® glucometer.

**Figure 7:**
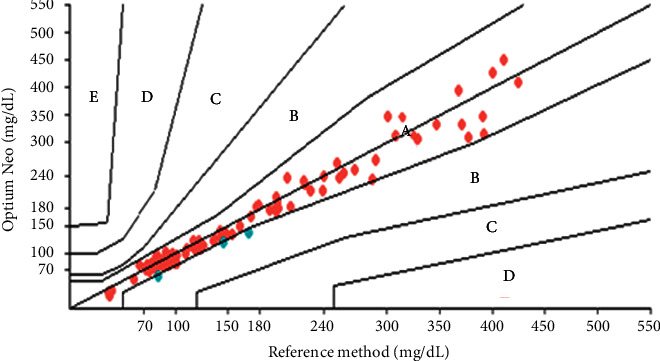
Error grid for the FreeStyle Optium Neo® glucometer.

**Figure 8:**
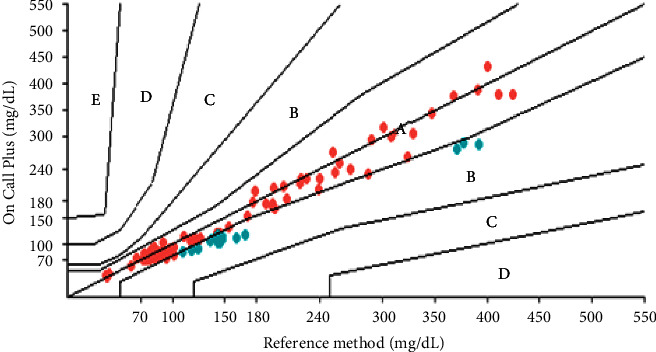
Error grid for the On Call plus® glucometer.

In the error grid analysis, all three glucometers obtained 100% of the results in zones *A* and *B*. The results obtained with the Optium Neo® glucometer had 97.39% of values in zone *A* and 2.61% in zone *B*. However, the On Call Plus® glucometer results were 86.09% in zone *A* and 13.91% in zone *B*. The analyses obtained with the Freedom Lite® glucometer were 19.13% in zone *A* and 80.87% in zone *B* (Table 6; Figures 6–8). To define a glucometer as precise, ISO 15197 : 2013 requires that 99% of the obtained results are in zones *A* and *B* [7] which was not observed in any of the glucometers tested herein.

The results obtained with the Optium Neo® glucometer reached the objective in four out of five analyses. In sequence, the On Call Plus® model reached a positive result in three of the five analyses. Finally, blood glucose measurements obtained using the Freedom Lite® glucometer were successful in only two of the five analyses (Table 7).

## 4. Discussion

Most portable glucometer blood glucose results are generally inferior to the results measured by the reference methods, and the higher the hyperglycemia, the greater the difference [3, 6]. In our study, all three glucometers underestimated the results of blood glucose concentration in relation to the reference method as well previously described in the literature [3, 7]. Analyzing the scatterplots, it was also observed that the higher the hyperglycemia, the greater the dispersion of the results in comparison with the reference method. The mean value for blood glucose concentration obtained by the reference method was 158 mg/dL. The FreeStyle Optium Neo® model showed less variation compared to the reference method (151 mg/dL), followed by the On Call Plus® model (145 mg/dL) and finally by Freedom Lite® (111 mg/dL). The three evaluated glucometers obtained a good correlation when compared with the reference method. Results from portable devices had a positive coefficient, showing that the values moved in the same direction compared to the reference method but does not attend the expected accuracy as previously discussed [7].

A study evaluated the FreeStyle Freedom Lite® model according to the ISO 15197 : 2013 guidelines [7] and reached 10% of results in the desired ranges. Regardless, our study showed that 7% of the results were in the established ranges. Regarding the error grid analysis, a study showed that Freedom Lite® glucometer results were 94% in zones *A* and *B* [7], and our results using the same model had 100% of values in zones *A* and *B* but with 80.87% of the analyses in zone *B*.

Until the conclusion of our study, there was no previous publication regarding the use of FreeStyle Optium Neo® and On Call Plus® models for dogs. However, previous models of the Optium product line have already been evaluated by some authors. On the Medisense Optium® model evaluation, a study analyzed the error grid and found that 78% of blood glucose concentration results were in zone *A*, 21% in zone *B,* and 1% in zone *C* (99% in zones *A*-*B*) [10]. Another study evaluated the Optium Xceed® model according to the ISO 15197 : 2013 guidelines and also performed an error grid analysis but failed to meet the established guidelines [7]. In such a study, 45% of blood glucose concentrations were in the established ranges, and when compared with our results using the Optium Neo® model, we obtained 92.2% of the results in the desired glycemic ranges. Regarding the error grid analysis, the literature shows that 99% of the results were in zones *A* and *B* with the Optium Xceed® glucometer [7], and using the Medisense Optium® model, another study observed 99% of the values in zones *A* and *B* [10]. In our study, when assessing the Optium Neo® model, 100% of the results were in zones *A* and *B* which do not differ from that previously described.

To define a glucometer as precise, the ISO 15197 : 2013 [13] standard recommends that 99% of the obtained results be in zones *A* and *B* [7]. All results obtained herein were in the *A* and *B* ranges (error grid). However, as the results in zone *B* deviate more than 20% in relation to the reference method, the authors recommend the use of glucometers that showed better performances in zone *A*, Optium Neo® (97, 39%) and On Call Plus® (86,09%).

Because of the type of sample used (venous, capillary, or arterial), there may be variations in the glucometer results [1, 2, 8], and different samples types could have been used. However, our study does not evaluate that since the main objective was to use only whole blood samples in comparison with the reference method. No glucometer inconsistency was identified during the calibrations, and the calibration results agreed with the manufacturer's instructions. A limitation of our study was the absence of reproducibility and repeatability analysis which measures the glucometer consistency in repeated tests. Since the Bland–Altman analysis was performed to determine the agreement between each glucometer and the reference method, we observed a significant bias, mainly for Freedom Lite. Then, another analysis was performed to explore such bias. The Passing Bablok results showed agreement between the glucometers and the reference method. These analysis discrepancies could have different hypotheses. One of them should be glucose measurement values >300 mg/dL where the discrepancy was more important when compared to <300 mg/dL (Table 3; Figures 3–5). Despite this divergence, Bland–Altman has more statistical power to detect than Passing Block [18]. The authors deny a conflict of interest with the manufacturers.

Portable human glucometers are widely commercially available and broadly used in veterinary practice. However, not all glucometers showed suitable for this purpose in our analysis. Therefore, the veterinarian must be attentive and use a glucometer validated for dogs' parameters. Regarding the evaluated glucometers, the FreeStyle Freedom Lite® glucometer had the worst performance herein, whereas the On Call Plus® glucometer achieved an intermediate performance, and the FreeStyle Optium Neo® model obtained the best results. Between the portable glucometers evaluated in our study, we concluded that On Call Plus® and FreeStyle Optium Neo® had a satisfactory glucose measurement in dogs and can be implemented in veterinary routines for easy and quick glucose evaluation purposes.

## Figures and Tables

**Table 1:** Blood glucose ranges in dogs from Brazil.

Blood glucose range (mg/dL)	Number of samples	Percentage (%)
<50	3	2.60
51–80	20	17.39
81–120	37	32.17
121–200	26	22.60
201–300	13	11.30
301–400	13	11.30
>400	3	2.60
Sample total	**115**	**100**

**Table 2:** Descriptive and comparative statistics of blood glucose concentration in different glucometers and the reference method in dogs from Brazil.

	M	MD	Min	Max	SD	25% perc	75% perc
Freedom Lite®	111^*∗∗*^	81	20	346	72	58	152
OptiumNeo®	151^*∗*^	110	20	478	98	82	203
OnCall Plus®	145^*∗*^	103	38	438	93	80	202
Laboratory	158	119	37	425	99	83	206

*Note*. *M*: mean; MD: median; Min: = minimum; Max: maximum; SD: standard deviation; Perc: percentile. ^*∗*^*p* > 0.05 in relation to the glucose oxidase reference method. ^*∗∗*^*p* < 0.05 in relation to the glucose oxidase reference method.

**Table 3:** Bland–Altman analysis of the three evaluated glucometers with the reference method in dogs.

Coefficients				
Glucometer	Bias	Std. deviation of bias	Agreement (CI 95%)	
Freedom Lite®	47,05	31,82	−25,39	119,49
Optium Neo®	6,23	17,08	−32,65	45,10
On Call Plus®	13,08	21,28	−35,36	61,52

**Table 4:** Percentage and absolute values of blood glucose concentration obtained using the three glucometers, according to the ISO 15197 : 2003 standards in dogs.

Established values	Freedom Lite			OptiumNeo			OnCall Plus		
	*n*	Total	%	*n*	Total	%	*n*	Total	%
<75 mg/dL: Difference of +/-15 mg/dL	4	9	44	9	9	100	9	9	100
≥75 mg/dL: Difference of +/- 20%	10	106	9.4	105	106	99.1	89	106	84.0
Total with inacceptable limits	**14**	**115**	**12.2**	**114**	**115**	**99.1**	**98**	**115**	**85.2**

**Table 5:** Percentage and absolute values of blood glucose concentration obtained using the three glucometers, according to ISO 15197 : 2013 standards in dogs.

Established values	Freedom Lite			OptiumNeo			OnCall Plus		
	*n*	Total	%	*n*	Total	%	*N*	Total	%
<100 mg/dL: Difference of+/- 15 mg/dL	8	50	16	48	50	96	49	50	98
≥100 mg/dL: Difference of +/-15%	0	65	0.0	58	65	89.2	42	65	64.6
Total within acceptable limits	**8**	**115**	**7.0**	**106**	**115**	**92.2**	**91**	**115**	**79.1**

**Table 6:** Error grid analysis of measurements obtained using glucometers.

Glucometer	Zone *A* (%)	Zone *B* (%)
Freedom Lite®	19.13	80.87
OptiumNeo®	97.39	2.61
OnCall Plus®	86.09	13.91

**Table 7:** Synthesis of the analysis of the effectiveness of glucometers in dogs' blood samples.

Glucometers	Descriptive/comparative	Correlations	ISO	ISO	Error grid
			2003	2013	
Freedom Lite®	No	Yes	No	No	Yes
OptiumNeo®	Yes	Yes	Yes	No	Yes
OnCall Plus®	Yes	Yes	No	No	Yes

Notes: NO: did not reach the goal; YES: reached the goal.

## Data Availability

The data used to support the findings of this study are available from the corresponding author upon request.

## References

[1] Wess G., Reusch C. (2000). Evaluation of five portable blood glucose meters for use in dogs. *Journal of the American Veterinary Medical Association*.

[2] Cohn L. A., Mccaw D. L., Tate D. J., Johnson J. C. (2000). Assessment of five portable blood glucose meters, a point-of-care analyzer, and color test strips for measuring blood glucose concentration in dogs. *Journal of the American Veterinary Medical Association*.

[3] Cohen T. A., Nelson R. W., Kass P. H., Christopher M. M., Feldman E. C. (2009). Evaluation of six portable blood glucose meters for measuring blood glucose concentration in dogs. *Journal of the American Veterinary Medical Association*.

[4] Domori A., Sunahara A., Tateno M., Miyama T. S., Setoguchi A., Endo Y. (2014). The clinical utility of two human portable blood glucose meters in canine and feline practice. *Veterinary Clinical Pathology*.

[5] Paul A. E. H., Shiel R. E., Juvet F., Mooney C. T., Mansfield C. S. (2011). Effect of hematocrit on accuracy of two point-of-care glucometers for use in dogs. *American Journal of Veterinary Research*.

[6] Nelson R. W., Feldman E. C., Nelson R. W. (2015). Canine diabetes mellitus. *Canine and Feline Endocrinology and Reproduction*.

[7] Brito-Casillas Y., Figueirinhas P., Wiebe J. C. (2014). ISO-based assessment of accuracy and precision of glucose meters in dogs. *Journal of Veterinary Internal Medicine*.

[8] Johnson B. M., Fry M. M., Flatland B., Kirk C. A. (2009). Comparison of a human portable blood glucose meter, veterinary portable blood glucose meter, and automated chemistry analyzer for measurement of blood glucose concentrations in dogs. *Journal of the American Veterinary Medical Association*.

[9] Mori A., Oda H., Onozawa E. (2016). Evaluation of portable blood glucose meters using canine and feline pooled blood samples. *Polish Journal of Veterinary Sciences*.

[10] Bluwol K., Duarte R., Lustoza M. D., Simões D. M. N., Kogika M. M. (2007). Avaliação de dois sensores portáteis para mensuração da glicemia em cães. *Arquivo Brasileiro de Medicina Veterinária e Zootecnia*.

[11] Ibm C. (2012). *IBM SPSS Statistics for Windows*.

[12] International Organization for Standardization (2003). In vitro diagnostic test systems—requirements for blood-glucose monitoring systems for self-testing in managing diabetes mellitus. European committee for standardization (CEN): brussels.

[13] International Organization for Standardization (2013). In vitro diagnostic test systems—requirements for blood-glucose monitoring systems for self-testing in managing diabetes mellitus. European committee for standardization (CEN): brussels.

[14] Parkes J. L., Slatin S. L., Pardo S., Ginsberg B. H. (2000). *Diabetes Care*.

[15] Clarke W. L., Cox D., Gonderfrederick L. A., Carter W., Pohl S. L. (1987). Evaluating clinical accuracy of systems for self-monitoring of blood glucose. *Diabetes Care*.

[16] Team R. C. (2017). R: A language and environment for statistical computing. https://www.R-project.org/.

[17] Schmolze D. (2017). Error grid analysis. https://cran.r-project.org/web/packages/ega/ega.pdf.

[18] Martin Bland J., Altman D. G. (1986). Statistical methods for assessing agreement between two methods of clinical measurement. *The Lancet*.

